# P-983. A Nationwide Survey of the Current Status of Antimicrobial Stewardship Programs in Korean Hospitals, 2024

**DOI:** 10.1093/ofid/ofaf695.1182

**Published:** 2026-01-11

**Authors:** Sung Min Lim, Raeseok Lee, Se Yoon Park, Jin Nam Kim, Ji Young Park, Choseok Yoon, Myung Jin Lee, Bongyoung Kim, Song Mi Moon, Hong-Bin Kim, Yong Chan Kim

**Affiliations:** Hanyang University Hospital, Hanyang University College of Medicine, Seoul, Seoul-t'ukpyolsi, Republic of Korea; Seoul St. Mary's Hospital, College of Medicine, The Catholic University of Korea, Seocho-gu, Seoul-t'ukpyolsi, Republic of Korea; Division of Infectious Diseases, Department of Internal Medicine, Soonchunhyang University Seoul Hospital, Seoul, Seoul-t'ukpyolsi, Republic of Korea; Hanyang University College of Medicine, soengdong-gu, Seoul-t'ukpyolsi, Republic of Korea; Department of Pediatrics, Seoul National University Children's Hospital, Seoul, Korea, Seoul, Seoul-t'ukpyolsi, Republic of Korea; Hanyang University Seoul Hosptial, Seoul, Seoul-t'ukpyolsi, Republic of Korea; Department of Internal Medicine, Inje University Sanggye-Paik Hospital, Seoul, South Korea, Seoul, Seoul-t'ukpyolsi, Republic of Korea; Department of Internal Medicine, Hanyang University College of Medicine, Seongdong-gu, Seoul-t'ukpyolsi, Republic of Korea; Seoul National University College of Medicine, Seoungnam-si, Kyonggi-do, Republic of Korea; Seoul National University Bundang Hospital, Seoul National University College of Medicine, Seongnam-si, Kyonggi-do, Republic of Korea; Department of Internal Medicine, Division of Infectious disease, Yongin Severance Hospital, Yonsei University College of Medicine, Yongin, Kyonggi-do, Republic of Korea

## Abstract

**Background:**

With growing global recognition of the importance of antimicrobial stewardship programs (ASP), the South Korean government launched a national ASP pilot project in November 2024 targeting hospitals with ≥300 beds. This study aimed to evaluate the status of ASP implementation at the initiation of the project.

**Figure:**
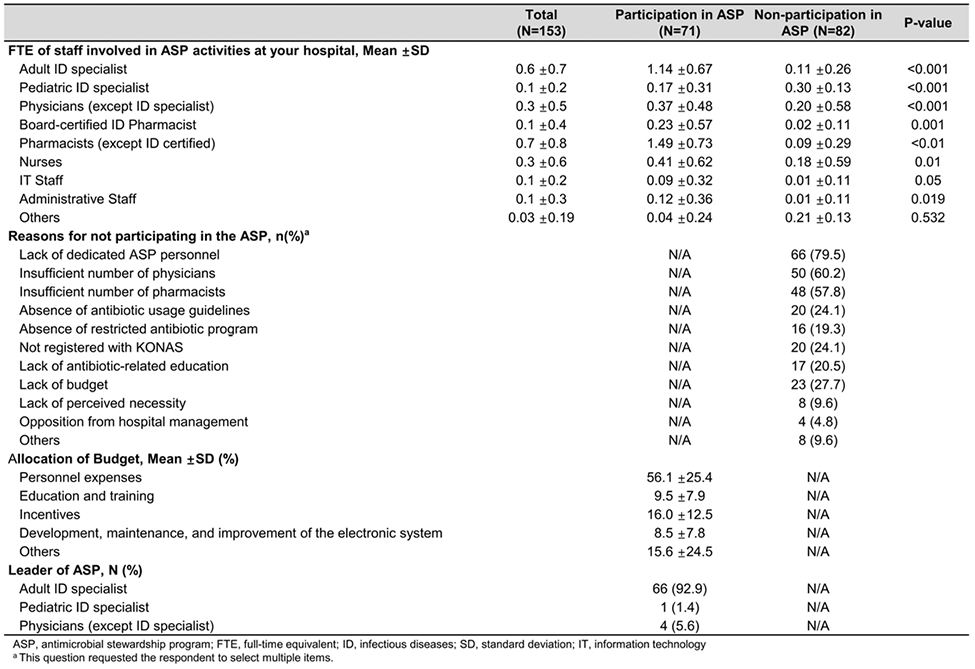
Human Resources and Composition of Antimicrobial stewardship program

**Figure:**
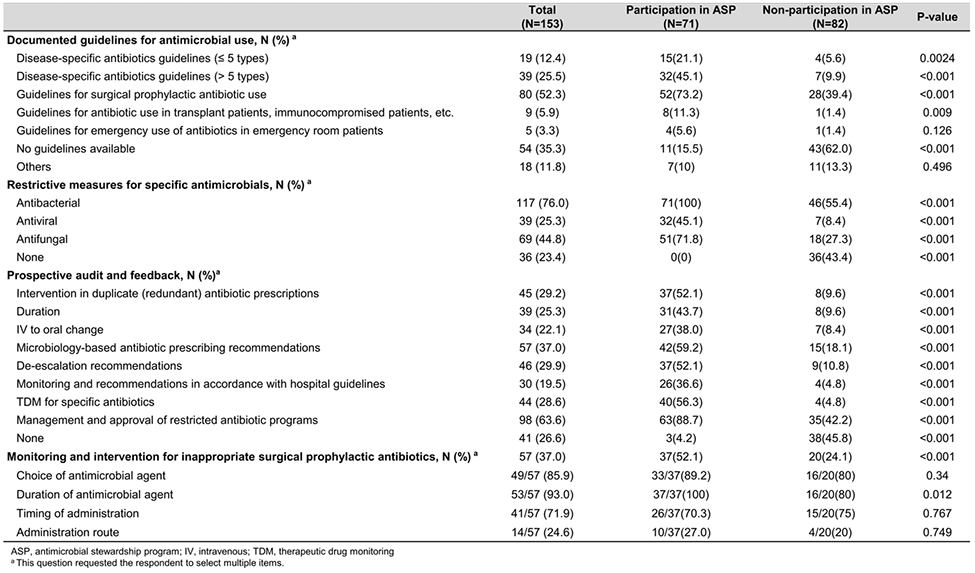
Activities for Antimicrobial stewardship program

**Methods:**

A nationwide survey was conducted from January to February 2025. The questionnaire, based on the 2020 survey, was updated through literature review and expert discussion. Hospitals with more than 300 beds were invited, and responses were collected from a single physician or pharmacist responsible for stewardship activities.

**Figure:**
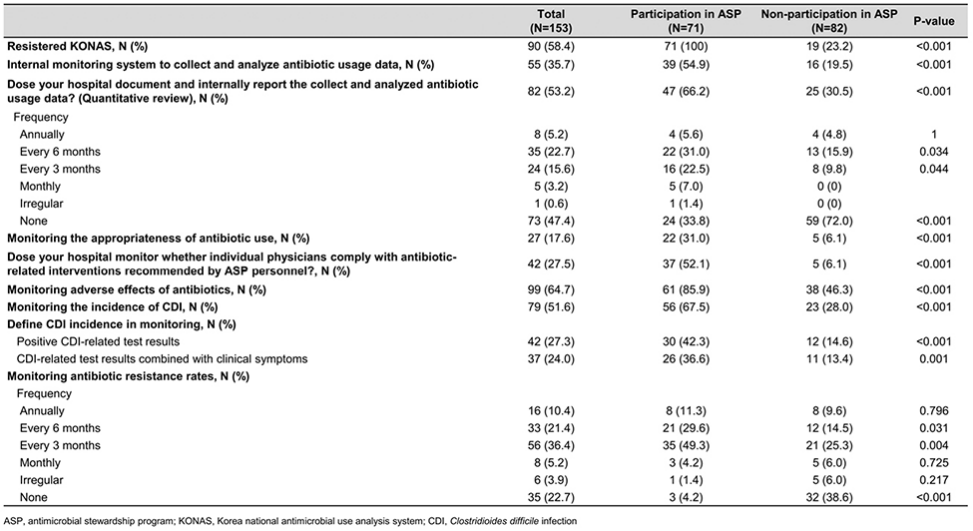
Monitoring and Reporting for Antimicrobial stewardship program

**Figure:**
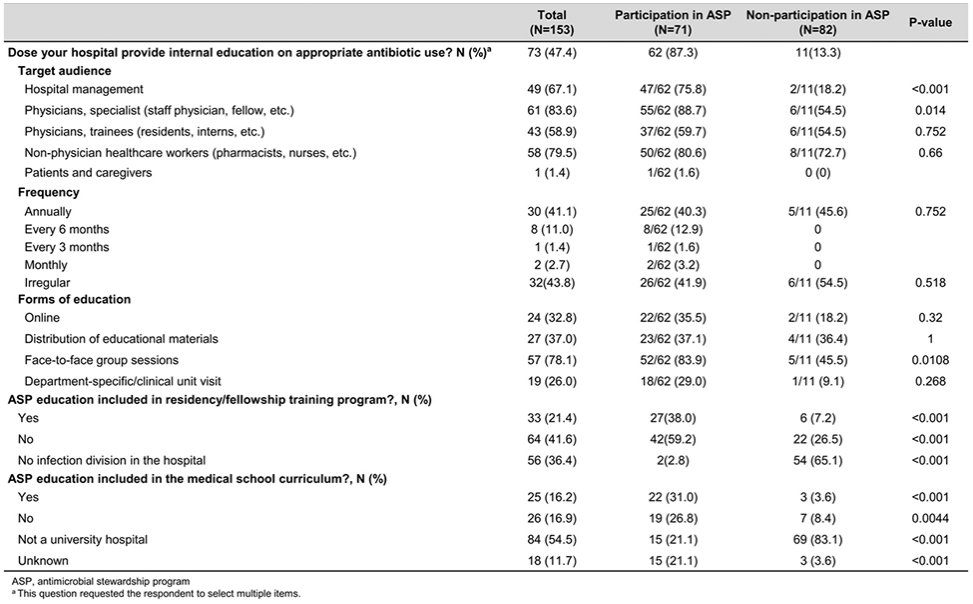
Education for Antimicrobial stewardship program

**Results:**

Of 297 hospitals, 153 (51.5%) responded. 71 hospitals (46.4%) participated in the ASP pilot project, while 82 (53.6%) were non-participating hospitals. The main reason for non-participation was a lack of dedicated personnel (79.5%). Among participants, 56.1% of ASP budgets were allocated to personnel costs and 16.0% to clinician incentives. ASP teams typically included general pharmacists (1.49 full-time equivalents [FTEs]), adult infectious disease physicians (1.14 FTEs), and nurses (0.41 FTEs). In ASP participating hospitals, pharmacists mainly conducted antibiotic use and resistance monitoring (93.0%), and nurses participated in ward rounds (33.8%).

Antibiotic guideline availability was 84.5% in ASP hospitals versus 38.0% in non-ASP hospitals. All ASP hospitals implemented restricted antibiotic programs, compared to 56.6% of non-participants. Active stewardship interventions such as microbiology-based interventions (59.2%), therapeutic drug monitoring (56.3%), and de-escalation of combination therapy (52.1%) were more common among ASP hospitals; these interventions were performed in fewer than 10% of non-ASP hospitals. Internal reporting of antibiotic use was performed by 66.2% of participants compared to 30.5% of non-participants. ASP-related education was conducted in 87.3% of participating hospitals versus 13.3% of non-participants.

**Conclusion:**

Hospitals that participated in the national ASP pilot project showed better management infrastructure and resource allocation. However, gaps also remain among participating hospitals, suggesting the need for ongoing support to achieve a successful ASP.

**Disclosures:**

All Authors: No reported disclosures

